# Transitioning from Microbiome Composition to Microbial Community Interactions: The Potential of the Metaorganism *Hydra* as an Experimental Model

**DOI:** 10.3389/fmicb.2016.01610

**Published:** 2016-10-13

**Authors:** Peter Deines, Thomas C. G. Bosch

**Affiliations:** Zoological Institute and Interdisciplinary Research Center, Kiel Life Science, Christian-Albrechts-Universität zu KielKiel, Germany

**Keywords:** microbiome, microbial interactions, synthetic communities, model system, systems biology, modeling

## Abstract

Animals are home to complex microbial communities, which are shaped through interactions within the community, interactions with the host, and through environmental factors. The advent of high-throughput sequencing methods has led to novel insights in changing patterns of community composition and structure. However, deciphering the different types of interactions among community members, with their hosts and their interplay with their environment is still a challenge of major proportion. The emerging fields of synthetic microbial ecology and community systems biology have the potential to decrypt these complex relationships. Studying host-associated microbiota across multiple spatial and temporal scales will bridge the gap between individual microorganism studies and large-scale whole community surveys. Here, we discuss the unique potential of *Hydra* as an emerging experimental model in microbiome research. Through *in vivo*, *in vitro*, and *in silico* approaches the interaction structure of host-associated microbial communities and the effects of the host on the microbiota and its interactions can be disentangled. Research in the model system *Hydra* can unify disciplines from molecular genetics to ecology, opening up the opportunity to discover fundamental rules that govern microbiome community stability.

## Introduction

Microbes sustain life on this planet as they perform not only important ecosystem functions but also inhabit all organisms. The entirety of a host with its associated microbial community, including viruses and cellular microbes is called the “metaorganism” or “holobiont” (Bosch and McFall-Ngai, 2011; Bosch and Miller, 2016). The existence of such a unifying term indicates the significance of the microbial community for understanding the biology of any host. These host-associated microbial communities (microbiomes) live on host surfaces, are associated with different tissues, and can reside inter- and intracellularly (Huttenhower et al., 2012; Kostic et al., 2013). Host-associated microbial communities are dynamic, changing throughout the hosts’ life, and are not passive players but actively engage in host development, metabolism, immunity, and health as found in established model systems, like corals, worms, insects, mice, and *Hydra* (Ley et al., 2008; Fraune and Bosch, 2010; Fukuda et al., 2011; Naik et al., 2012; Lee and Brey, 2013; Sommer and Bäckhed, 2013; McFall-Ngai, 2014; Thompson et al., 2015). How microbes increase the host’s stress tolerance and modulate its niche breath are active fields of research (Mueller and Sachs, 2015). Despite the evident importance of the microbiome in affecting host fitness (in a Darwinian sense), insights into the underlying mechanisms are still lacking.

## Transitioning from Descriptive to Predictive Microbiome Research

Recent technological advances (e.g., high-throughput sequencing, proteomics, metabolomics) and expanded efforts through a large number of human and environmental microbiome initiatives (summarized in Stulberg et al., 2016) have led to great progress in characterizing the composition of host-, habitat-, or ecosystem-associated microbial communities (Huttenhower et al., 2012; Gilbert et al., 2014; Hacquard et al., 2015; Sunagawa et al., 2015). A recent assessment of US microbiome research, for example, highlights the importance of microbiome studies for tackling current world problems, such as food production, human, and ecosystem health (Stulberg et al., 2016). Yet for achieving this, microbiome research needs to shift from a descriptive to a more predictive science (Alivisatos et al., 2015), where the ecology of these highly diverse communities is addressed. Central for the ability to predict and manage the function of host-associated microbial communities is the knowledge about the factors determining their dynamics and stability. The concept of a core microbiome (taxa or functional core) has been very helpful in addressing the stability of this core and how it changes with age, diet, geographic location, time, or other factors. Recent findings from human microbiome research suggest that a core microbiome can be defined at the functional rather than the taxonomic level (Lloyd-Price et al., 2016). Yet, what constitutes a core still remains elusive and depends on the question of interest. In *Hydra*, microbial communities of wild caught and domesticated animals have been found to be surprisingly similar and to share a core microbiota at the taxonomic level (Fraune and Bosch, 2007). It is likely that the microbiome (like microbial communities associated with abiotic environments) is affected by various extrinsic and intrinsic factors, e.g., temperature, pH, resource availability, microbe–microbe interactions, but also by interactions with the host. While a number of studies exist on the interactions *between* host and the microbiome, comparatively little is known on the interactions between microbes *within* the microbiome (including the virome) and on how these impact the metaorganism.

**FIGURE 1:**
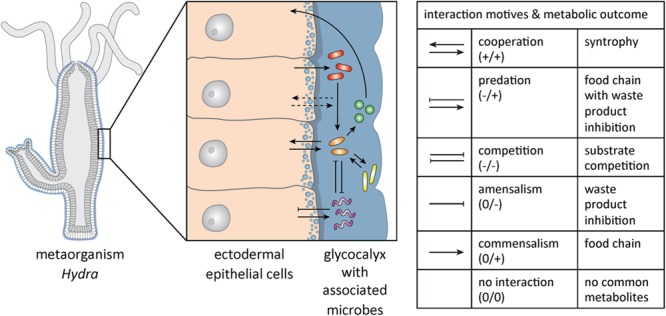
**Complex microbial community interactions in *Hydra.*** Schematic drawing of *Hydra*, with a magnified section of the ectodermal epithelial cells that are covered with a glycocalyx and the associated microbial community. In this complex system microbes may interact with each other in multiple ways as depicted by arrows. For the species involved interactions can have a positive (+), a negative (-) or no impact (0). A summary of the possible ecological interactions and their meaning in metabolic terms are summarized in the table (modified from West et al., 2007; Faust and Raes, 2012; Großkopf and Soyer, 2014). It is likely that interactions between specific microbes and the host can be modulated by metabolic or physical signals but that the host also directly affects the interaction between microbial species (dashed arrows) via epithelial selection.

Within-microbiome interactions can be driven by diverse features such as metabolism, social traits (production of public goods), or environmental factors, like spatial organization (Kim et al., 2008; Nadell et al., 2010; Mitri and Foster, 2013; Großkopf and Soyer, 2014). Six different interaction patterns between members of different species can be distinguished (Lidicker, 1979) (see **Figure 1** for potential interactions within the metaorganism exemplified in *Hydra*).

## Microbe–Microbe Interactions: Challenges and Future Research

For disentangling the interactions in microbial communities, it is essential to understand the importance of the two key motifs, cooperation and competition. This has been well studied in environmental communities, e.g., by Foster and Bell (2012), where species from two aquatic environments were isolated. By analyzing pair-wise interactions between the culturable microbes it was observed that the majority of interactions were competitive (Foster and Bell, 2012). This pattern is confirmed by results from other studies, which suggest that at least 86% of interactions between strains or species are competitive (reviewed in Mitri and Foster, 2013). This is in contrast to what had been assumed for host-associated microbial communities until very recently. Cooperative interactions among community members were predicted to be the major driving force for a productive and stable microbiome, e.g., in the human gut (Bäckhed et al., 2005; Van den Abbeele et al., 2011). This view was challenged by a seminal study that integrated ecological network analysis and recent data from mammalian microbiomes (Coyte et al., 2015). Whereas a cooperative microbial network was indeed found to be very productive in the short term, competition between the different microbes was identified as the driver of long-term microbiome stability, and so as a benefit for the host. The counter-intuitive result that cooperation between microbial species is destabilizing is based on positive feedback loops that will lead to runaway effects (Coyte et al., 2015). Unconstrained cooperation is predicted to lead to an ever-increasing abundance of the cooperating groups of species, which in turn can result in the collapse of competing populations and eventually in the destabilization of the whole community. Competitive interactions between microbes are in contrast thought to help stabilize the community (McNally and Brown, 2016).

Another factor facilitating the stability of its microbiome is the host itself (Coyte et al., 2015). Several mechanisms have been identified by which a host may be able to suppress the positive feedback between cooperating species and weaken their interaction: (i) regulation through the immune response dependent on the density of the different microbial species, (ii) spatial segregation reducing between-species contact, and (iii) provision of carbon sources via epithelial feeding minimizing cross-feeding between microbes. This study also implies that for understanding and manipulating the host microbiome, close attention needs to be paid to the strength and nature of the ecological interactions between the different microbial species (Coyte et al., 2015), for which experimental data is still scarce.

Although impressive advances have been made with the help of high-throughput sequencing techniques in describing the (highly) diverse species compositions of host-associated communities [such as the ‘Human Microbiome Project’ (Huttenhower et al., 2012)], little data is currently available on ecological interactions within the microbiome (Coyte et al., 2015). One central aspect for approaching a predictive understanding of microbial community function and dynamics will be the integration of theory and experiments (Widder et al., 2016). For generating data that can feed into mathematical models, ‘model’ microbial communities need to be identified that can be manipulated and where theoretical predictions can be tested.

## *Hydra* and Its Microbiome as an Experimental Model

We here propose to extend the utilization of the metaorganism *Hydra* beyond the fields of developmental biology (Bosch, 2007), stem cell research (Bosch, 2009; Bosch et al., 2010), immunity (Bosch, 2013, 2014), aging (Nebel and Bosch, 2012; Boehm et al., 2013), and animal–microbe interactions (Bosch, 2013, 2014) as a model organism for studying interactions *within* the animal-associated microbiota and on how that affects the host and vice versa (**Figure 1**).

*Hydra* is a cnidarian, which in contrast to other model systems for host-associated microbiota, is phylogenetically basal. *Hydra’s* phylogenetic position thus provides the benefit of a very simple body plan with a limited number of cells and a basal immune and nervous system. Its tube-like body is akin to the vertebrate intestine (Bosch, 2012) and changes in *Hydra’s* epithelial homeostasis lead to significant changes in the microbial community (Fraune et al., 2009). *Hydra* possesses a species-specific and core microbiome of low complexity (Fraune and Bosch, 2007; Bosch, 2013), from which the most dominant microbes can individually be cultured under laboratory conditions (Bosch, 2013; Fraune et al., 2014). The observation of distinct and reproducible colonization patterns of *Hydra* during its developmental life cycle suggests that host factors are involved in shaping the microbial composition (Franzenburg et al., 2013a). Antimicrobial peptides have been identified as prominent effector molecules of the innate immune system that drive epithelial selection (Augustin et al., 2009; Bosch et al., 2009; Franzenburg et al., 2013b). Yet it is unlikely that the host has the ability to control each microbial phylotype individually.

Another key tool for studying interactions between the host and its microbiota is the creation of germ-free or gnotobiotic animals, which has been achieved in the *Hydra* system (Franzenburg et al., 2012). This in combination with its transparency, its fast life cycle, the ease of its cultivation and clonal propagation, and the rich pool of knowledge on features important for characterizing, understanding, but also manipulating *Hydra* as a host (for details see Bosch, 2014), makes it a perfect system for ‘deconstructing’ a metaorganism and its interactions. *Hydra* as a basal eumetazoan thus not only allows us to gain insight into the early evolution of host–microbe interactions but also into the ecological interactions within low complexity microbiomes. Dissections of the ecological interactions at play are key for understanding microbiome dynamics, which is the prerequisite for the ability to reconstructing and restoring a ‘healthy microbiome’.

**FIGURE 2:**
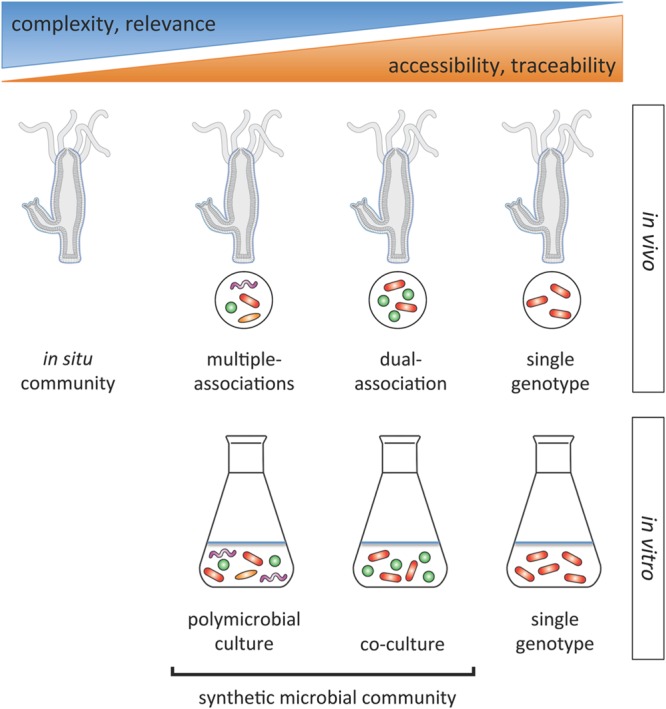
***Hydra’s* experimental potential for studying interactions within the microbiome.** Overview of the spectrum of microbial community study systems for unraveling interactions in the relatively simple *Hydra* microbiome. The integration of *in vivo* and *in vitro* experiments offers the opportunity to capture clues on how the host might affect microbe–microbe interactions and vice versa. The opportunity to study inter- and intra-specific interactions at different resolutions will help to unravel general principles of microbial interactions in microbiomes.

For disentangling microbial interactions within the *Hydra* microbiome, we propose an integrated approach based on constructing synthetic communities of various complexities *in vivo* and *in vitro* that can be compared to the *in situ* community and to single genotypes (**Figure 2**). Contrasting identical microbial communities through *in vivo* and *in vitro* experiments will offer valuable clues to the extent of host effects on microbe–microbe interactions and ultimately their fitness, and vice versa of microbial effects on the fitness of the host. Classical co-culture experiments (Mitri and Foster, 2013) can easily be performed in the *Hydra* system. Here the community is deconstructed to its individual components and fitness of each microbial species within the synthetic communities of various compositions compared to its fitness when grown singly. Negative interactions [such as competition (net negative effect)] result in reduced growth of the co-culture compared to the sum of the species (mono-culture) yields. If additive growth equals the sum of mono-culture growth, the net effect is neutral, so species do not interact. If the net effect is positive, i.e., the combined growth is greater than the additive growth, interactions are cooperative. This approach has been used for species assemblages with a manageable number of species (e.g., Fiegna et al., 2015). With the ability to culture most of the microbial phylotypes in *Hydra* *in vitro* (Bosch, 2013), the interaction between individual members and the role of individual microbes within the community can be disentangled.

Co-culture experiments will provide information on the interactions between the different microbes but also on the interactions with the host (**Figure 1**), which can be implemented into simple theoretical models that can in turn be extended to providing predictions for more complex microbial communities, such as the mouse or human microbiome. The value of such an approach in gaining new insights into the function of the metaorganism has been showcased by a recent study, where *Hydra’s* microbiome was found to provide protection from infection with the fungus *Fusarium* sp., which was only able to infect germ-free animals or the ones with a reduced microbial community (Fraune et al., 2014). Data from these experiments fed into theoretical models, which confirmed the findings using a game theory approach, but also indicated that more experiments are needed, as the findings could not fully be explained by pairwise interactions between the microbial species (Li et al., 2015). Such integration of mathematical predictive models with experimental data is essential for advancing the understanding of the function and dynamics of microbial communities (Widder et al., 2016).

For gaining information on the mechanistic underpinning of specific interactions, co-culture experiments can be complemented with gene knockout experiments (Bernstein et al., 2012; Nakashima and Miyazaki, 2014; Khare and Tavazoie, 2015) in which a target gene is deleted in one of the strains and the nature of the interaction in the co-culture compared to the one with the gene still present. Rakoff-Nahoum et al. (2016) used such an *in vitro* approach in combination with gnotobiotic mouse experiments to test for the evolution of cooperation within the mammalian gut microbiota. Further, there is evidence that not only genetic changes might be necessary for the establishment and maintenance of interspecies interactions but also that changes of the transcriptome can solely be sufficient (reviewed in Tan et al., 2015). The opportunity of analyzing the mechanistic (genetic) underpinning of the interactions among microbes is possible in the *Hydra* metaorganism but can moreover be extended to the host as not only the bacteria can be genetically manipulated, but also transgenic *Hydra* polys can be generated by embryo microinjection (Wittlieb et al., 2006). Another aspect that is advantageous in this system is the ability to create a ‘static’ host that doesn’t coevolve with the microbiome as *Hydra* can reproduce asexually via budding, and so create identical copies of itself. The microbiome in contrast can artificially be manipulated and selected upon, e.g., with the goal of improving host performance. This host-mediated microbiome engineering (recently reviewed in Mueller and Sachs, 2015) is discussed as one of the new research frontiers in medicine and agriculture. Microbiome engineering also allows the studying of the emergence of new interactions between community members and how this affects the productivity and long-term stability of the microbiome and also on how it impacts on host fitness (Mueller and Sachs, 2015). This is especially of interest in times of rapid environmental change, where shifted interactions within the microbiome can function as a buffer against environmental effects on the host.

Using host-mediated microbiome engineering, another layer of complexity can be added by including the virome. A recent study revealed that viral communities in *Hydra* are species-specific (Grasis et al., 2014) but their role in establishing and maintaining the microbiome or affecting species interactions has to be still resolved (Bosch et al., 2015; Bosch and Miller, 2016).

Decoding the connection between metabolites, microbes, and the host is yet another exciting frontier in metaorganism research (Dorrestein et al., 2014). This is highly relevant in the light of recent evidence that microbially produced metabolites influence different organ systems, such as microbe-brain connections, bone metabolism, or immune functions (Cryan and Dinan, 2012; Dorrestein et al., 2014; Charles et al., 2015; Eisthen and Theis, 2016). Multi-omics approaches in which the metabolome, the microbiome, and the host immune system are assessed simultaneously are feasible in the *Hydra* model and will help to decipher this new connection. The metabolite exchange between the different microbial players in an interaction can be addressed in an experimental set up, where a conditioned- or spent-medium approach allows to control metabolite production and consumption (Ponomarova and Patil, 2015). The method is based on a cell-free culture filtrate of a donor species, which is added to a recipient microbial culture to assess the activity of the secretome (Vetsigian et al., 2011; Rakoff-Nahoum et al., 2014). In addition to classical experiments in liquid culture media, other co-culture systems and technologies are currently used, such as microfluidics, petri dishes, solid support systems, bioreactors, and transwell systems (reviewed in detail in Goers et al., 2014).

An alternative approach for inferring cooperative and competitive relationships between the different community members (but not the underlying mechanisms) is based on the availability of high-throughput sequence data, where co-occurrence data and correlation patterns are analyzed (Faust and Raes, 2012). A positive relationship can be assumed, when two species co-occur or show a similar abundance pattern over multiple samples; a negative one is predicted, when they show mutual exclusion (Faust and Raes, 2012). With this information co-occurrence interaction networks can be constructed, enabling the identification of keystone species that stabilize a community, or predicting the (in)stability of communities to environmental change (Faust et al., 2012; Berry and Widder, 2014; Widder et al., 2014).

Another set of models based on whole-genome sequence data (or at least drafts) focuses on metabolic dependencies in microbial communities (Klitgord and Segrè, 2010; Mahadevan and Henson, 2012). Based on the success of systems biology in developing *in silico* predictive capabilities for individual species, community systems biology (CoSy) attempts to determine and predict the interactions among multiple species (Zengler and Palsson, 2012; Tan et al., 2015). The role of community systems biology in helping to unravel modes of interactions in complex communities has recently been highlighted by Zengler and Palsson (2012), and specifically for the human microbiome by Manor et al. (2014). Genome-scale metabolic modeling has been instrumental for the reconstruction of the genotype to phenotype relationship for single organisms (Borenstein et al., 2008; Zengler and Palsson, 2012). The advancement of these frameworks and techniques for multispecies metabolic models is a very promising route for the prediction of complex relationships in multispecies systems (such as the human microbiome) in the near future (Borenstein, 2012; Manor et al., 2014). Such an approach can be applied to the *Hydra* system due to its comparatively simple microbiome. Hypotheses can be generated based on genomic data for subsequent tests in the laboratory as most of the bacterial phylotypes identified can be cultured *in vitro*.

The ease of working with the *Hydra* system in the laboratory allows that experiments can be performed under well-controlled conditions, that can easily be replicated, perturbed, and sampled at different time scales. *Hydra* shares many ancestral genes with humans that have been lost in *Drosophila* and *Caenorhabditis* (Chapman et al., 2010; Hemmrich et al., 2012), so insights into host–microbe–microbe relationships might unravel general principles that are also relevant to humans and their microbiota. The *Hydra* system provides an excellent bridge between the simplicity of synthetic communities and the mouse model. As important features of the host, e.g., the host immune response, are difficult to recapitulate *in vitro*, *in vivo* experiments in combination with an *in silico* approach will close the knowledge gap from microbiome composition to ecological interactions within these communities.

## Author Contributions

PD and TB jointly contributed to conception and writing of the manuscript. Both have approved it for publication.

## Conflict of Interest Statement

The authors declare that the research was conducted in the absence of any commercial or financial relationships that could be construed as a potential conflict of interest.
